# Prism adaptation combined with serious games for improving visual-constructive abilities in stroke patients: randomized clinical trial

**DOI:** 10.3389/fdgth.2025.1425410

**Published:** 2025-02-21

**Authors:** Massimiliano Oliveri, Sergio Bagnato, Silvia Rizzo, Emilia Imbornone, Patrizia Turriziani

**Affiliations:** ^1^ Department BIND, University of Palermo, Palermo, Italy; ^2^ NeuroTeam Life and Science, Palermo, Italy; ^3^ Restorative Neurotechnologies, Palermo, Italy; ^4^ Villa Rosa Rehabilitation Hospital, Provincial Agency for Health Services (APSS), Pergine Valsugana, Trentino, Italy; ^5^ Rehabilitation Department, Unit of Neurophysiology and Unit for Severe Acquired Brain Injuries, Giuseppe Giglio Foundation, Cefalù, Italy; ^6^ Department SPPEF, Neuropsychology Lab, University of Palermo, Palermo, Italy

**Keywords:** prism adaptation (PA), stroke, serious games (SG), rehabilitation, neuropsychology, apraxia, neglect

## Abstract

**Introduction:**

Visuomotor adaptation to a displacement of the visual field induced by prismatic lenses can help rehabilitate cognitive deficits when combined with digital cognitive training. The aim of this study was to evaluate the effectiveness of this approach in rehabilitating visual constructive deficits in stroke patients, assess the generalization of improvements to daily living skills, identify which serious games best predicted improvements.

**Methods:**

Thirty stroke patients were randomly assigned to either a control group, receiving standard rehabilitation, or an experimental group, receiving a therapy combining prism adaptation with cognitive training through serious games over ten consecutive sessions. Patients were administered a neuropsychological test battery at baseline (T0) and after 10 days (T1). Visual constructive abilities were evaluated using Freehand Copy of Drawings and Copy of Drawings with Landmarks tests. Spatial attention was evaluated using Albert's Line Cancellation and Line Bisection tests. Functional abilities were evaluated with the Barthel Index.

**Results:**

Test scores of the Freehand Copy of Drawings improved from T0 to T1 in both the experimental (6.89 ± 2.7 vs. 7.83 ± 2.9; *p* = 0.01) and the control group (5.84 ± 2.1 vs. 7.51 ± 2.2; *p* = 0.01). The improvement was comparable between the two groups (*p* = 0.38). Test scores of the Copy of Drawings with Landmarks improved from T0 to T1 in the experimental (42.94 ± 19.6 vs. 50.2 ± 18.1; *p* = 0.007), but not in the control group (39.9 ± 19.6 vs. 42.7 ± 20.9; *p* = 0.41). The improvement was comparable between the two groups (*p* = 0.28). In the experimental group, Barthel Index scores at T1 correlated with both Free Hand Copy of Drawings scores (*R* = 0.651; *p* = 0.009) and Copy of Drawings with Landmarks scores (*R* = 0.582; *p* = 0.02). No correlations were found in the Control Group. Serious games targeting attention and motor planning were predictive of improvements in visual construction.

**Conclusion:**

prismatic lenses combined with digital cognitive training improve visual construction and functional abilities in stroke patients, providing a novel method to promote stroke rehabilitation.

## Introduction

Visual construction refers to the ability to assemble the elements of 2- or 3-dimensional objects while maintaining their orientations and spatial relationships. This ability can be evaluated through figure-copying tasks (1, 2). Impairment of this ability is called constructional apraxia, it can result from unilateral brain injury, affecting either the right or left hemisphere, and it has a prevalence ranging from 15%–40% of stroke patients (3–5). Deficits in visual constructive abilities are linked to various dysfunctional components, including visuospatial skills, executive functions, and language (1, 6).

Stroke patients with visual constructive deficits often experience difficulties in performing daily living activities (ADL) (7). In particular, they may struggle with tool use, which can lead to safety concerns following discharge from acute hospital care (6, 8).

From a rehabilitation perspective, despite the high incidence of apraxia and its significant impact on ADL, a common clinical approach has been to adopt a wait-and-see strategy, assuming that apraxia will recover spontaneously (9). However, research shows that 88% of apraxic patients in the acute stage remain apraxic 20 weeks after initial assessments, particularly those with mild impairments (10). As a result, rehabilitation for apraxia remains a major challenge for clinicians and occupational therapists.

The generalizability of rehabilitation outcomes, especially regarding their broader impact on functional independence, is often either not assessed or not well documented in the literature (11). In the case of visual constructive deficits, to facilitate generalization it is crucial to develop technology-driven solutions that can address the multiple factors underlying these deficits, such as visuospatial attention, visuomotor coordination, semantic processing, and motor planning.

A recent innovation in post-stroke rehabilitation is the development of a digital device designed to address cognitive impairments. The Mindlenses Professional device combines prismatic goggles, which induce a 10° deviation of the visual field to the right or left, with a platform of serious games aimed at training visual attention and executive functions.

During the visual field deviation induced by the prismatic goggles, the patient is instructed to make pointing movements towards visual targets presented on a tablet. After a few trials, the patient adapts to the visual distortion and can correctly point to the targets: the process is called prism adaptation [PA; see (12) for an example of its application in stroke patients].

Previous studies have shown that visuomotor adaptation to prisms is associated with increased cortical excitability in the hemisphere ipsilateral to the visual field deviation, an effect comparable to that induced by non-invasive brain stimulation techniques (13). This suggests that prism adaptation might serve to “prime” a brain network to make it more susceptible to a cognitive-behavioral intervention that follows neuromodulation, as already tested with prior TMS [e.g., (14)] and tDCS [e.g., (15)] studies.

Consistent with this hypothesis, the combination of prism adaptation followed by cognitive training through serious games has proven effective in rehabilitating cognitive deficits in stroke patients, with improvements correlating with better performance in daily living activities (16).

The present study aimed to test the efficacy of this digital visuomotor and cognitive stimulation approach for treating visual-constructive deficits in stroke patients.

The rationale behind this approach was to enhance cortical excitability in the stroke-affected hemisphere through prism adaptation and then to leverage this cortical neuromodulation with digital cognitive training targeting fundamental processes involved in visual constructive abilities, such as sustained attention, semantic analysis, and planning. Additionally, we sought to assess the impact of this kind of rehabilitation on daily functional activities and to identify which functional components, addressed by specific serious games, are most closely associated with improvements in visual-constructive tasks.

## Patients and methods

The study was conducted in the neurorehabilitation unit of Ospedale Giglio in Cefalù (Italy). The trial was approved by the Ethical Committee of Palermo 1 (n◦ 06/19) and it was conducted in compliance with the Declaration of Helsinki. Trial was retrospectively registered in the ISRCTN registry (number: ISRCTN12243194).

### Study design and participants

Thirty consecutive patients with first ever unilateral ischaemic or hemorrhagic stroke were recruited during the subacute phase. The sample size was estimated on the basis of the magnitude of the effects of prism adaptation on cognitive functions (i.e., temporal perception) as tested in previous studies in stroke patients. Power analysis with alpha level of 0.05 and beta of 80% was applied.

Details of the patients' demographic and clinical characteristics are reported in Table 1.

**Table 1:** Clinical and demographic characteristics of recruited patients.

	Prisms + Serious games Group	Control Group
Age (years)	65.1 ± 14.6	56.9 ± 12.4
Gender (F/M)	8/7	6/9
Education (years)	5.9 ± 3.2*	13.7 ± 3.6
Time since the stroke (days)	53.5 ± 29.6	53.9 ± 29.7
Ischaemic (*N*)	10	6
Haemorragic (*N*)	5	9
Right Brain Damage (*N*)	10	13
Left Brain Damage	5	2
Neglect	4	8

* *p* < 0.05.

Randomization of the patients in an experimental and a control group was made using randomization with a 1:1 allocation, by referring to an online random number generator (graphpad.com) that randomly scrambled 30 participants among a 2 treatment slots, so that each treatment always got assigned the same number of participants.

Randomization was made by a clinician not involved in the subsequent clinical study phases. Therefore, the allocation sequence was concealed to personnel involved in interventions until participants were enrolled and assigned to interventions.

Patients of the control group received a routine cognitive rehabilitation program, with 10 consecutive sessions distributed over two weeks. Patients of the experimental group received an experimental training using the medical device Mindlenses Professional that integrates PA and serious games. The protocol lasted 10 sessions and it was distributed over two weeks.

Both control and experimental groups additionally received daily physiotherapy and occupational therapy. Patients with visual-constructive deficits in the control group were treated with copy of drawings exercises.

The clinician collecting the data could not be blinded to groups since the treatment of the experimental group was clearly distinguishable from that of the control group.

**Figure 1:**
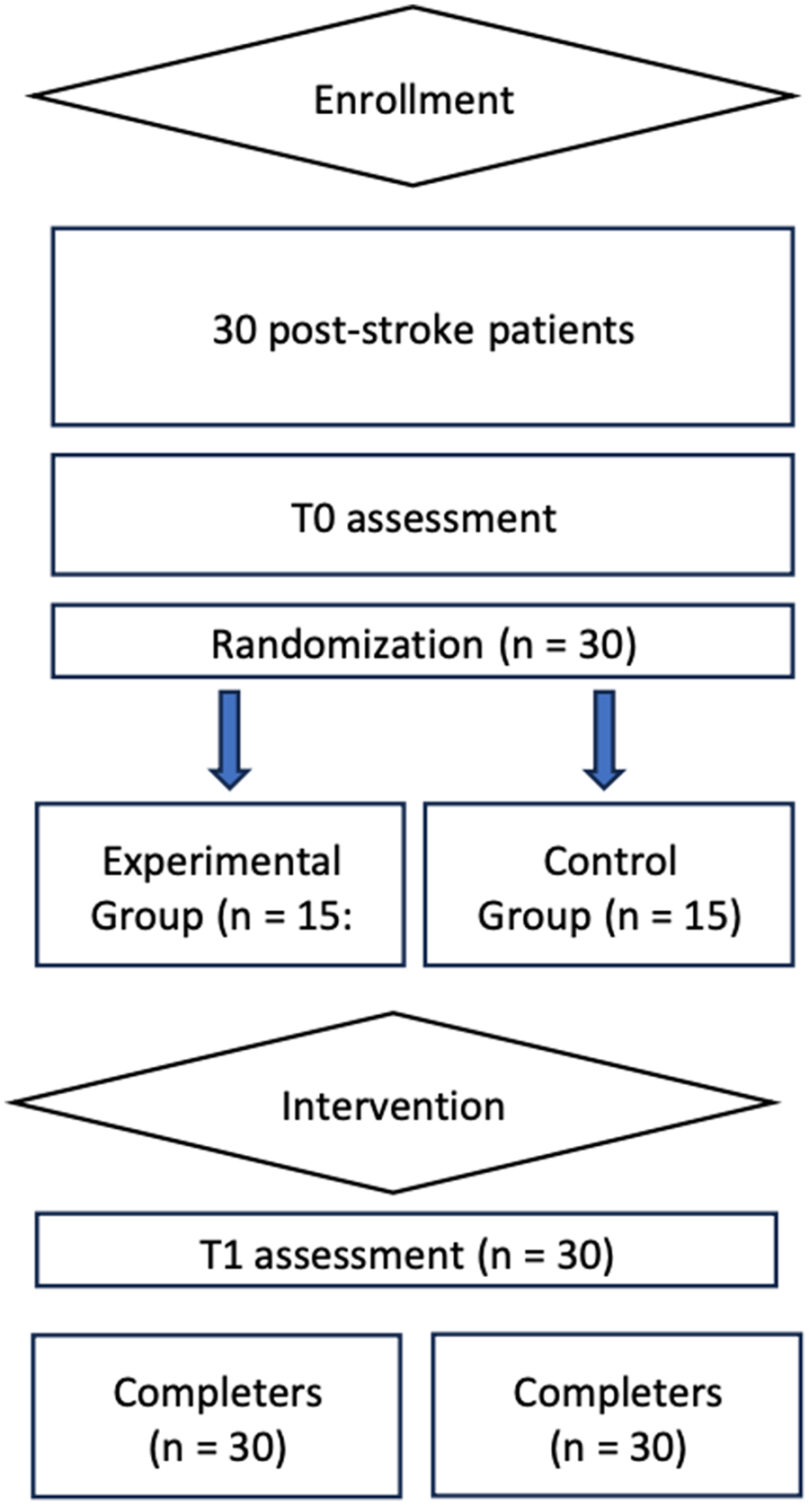
Study flow chart.

Figure 1 shows the study flow chart. All patients of the experimental and the control group completed the study and there were no drop-outs.

### Experimental intervention

In the experimental group, each training session started with a digitized PA session. Patients wore prismatic lenses with a power of 20 dioptries. According to previous neurophysiological findings, showing an increase of cortical excitability in frontal areas of the hemisphere ipsilateral to prismatic deviation (17), right-brain-damaged (RBD) patients were exposed to rightward prisms and left-brain-damaged (LBD) patients to leftward prisms.

The adaptation procedure was digitized, using an 11″ tablet, positioned at a distance of 53 cm from the patients' eyes and with its center aligned to the patient's sagittal midplane, for random presentation of visual targets (square dots subtending 1° of visual angle) in one of three spatial positions of the tablet's screen: in the center of the screen or lateralized to the right or the left space (21° of visual angle). Patients had to point at a fast but comfortable rate towards the visual targets using their ipsilesional hand. The visual targets persisted on the screen until patients' touch or disappeared after a fixed interval of 1 s

In the pointing procedure, 60 targets were randomly presented. Following this phase, the goggles were removed, and the patient started the cognitive training session with seven serious games. The procedure (prism adaptation followed by seven serious games) was repeated for ten consecutive sessions (i.e., five days a week per two consecutive weeks).

For each of the ten sessions, following the termination of the prism adaptation phase, seven serious games were consecutively administered in this order: “Beware of the bomb”, targeting sustained attention and decision making; “Visual search”, targeting simple and conjunction visual search; “The café”, targeting divided attention, planning and switching; “Go No Go”, targeting inhibition; “Reverse order”, targeting visuo-spatial attention and spatial working memory; “Semantic associations”, targeting visual semantic associations; “Calculation”, targeting calculation and verbal working memory. Details of the serious games are reported in Oliveri et al. (16).

All games have been implemented using a dynamic difficulty algorithm, i.e., adapting the speed of stimulus presentation and the game difficulty to the single patient's performance. A total score is calculated for each game in each session. According to the dynamic difficulty algorithm, higher scores are automatically assigned by the software to correct answers at higher difficulty levels while lower scores are assigned to correct answers at lower difficulty levels.

All patients were tested with a battery of neuropsychological tests including measures of attention, executive functions, memory, visuospatial abilities, and language. In particular, the following tests were administered: Oxford Cognitive Screen for general cognitive functioning (18); Raven's coloured progressive matrices for non-verbal intelligence and reasoning (19) VOSP for visual perception (20); Attentional Matrices (21), Line Bisection (22) and Albert's line cancellation (23) tests for visual and spatial attention; Stroop test (24), Digit and Spatial Span backward (25), and Phonological fluencies (19) for executive functions; Digit and Spatial Span forward (25), Rey Auditory Verbal Learning Test (19) and recall of the Rey-Osterrieth Complex Figure (26) for learning and memory domain; copy of the Rey-Osterrieth Complex Figure (26) for the visuospatial domain; Freehand copy of drawings and copy of drawings with landmarks for the visual-construction domain (19); Semantic fluencies (21) and BADA (27) for the language domain; Tests for Ideomotor and Buccofacial Apraxia (21), for the apraxia domain.

The Barthel Index (28) was used for functional analysis of activities of daily living.

Tests were applied at T0 (before the control or the experimental training), and at T1 (after the 10 training sessions).

We report here the results regarding the tasks of Freehand copy of Drawings, Copy of drawings with landmarks, Albert's line cancellation test and Line bisection test. All results have been previously published in the ISRCTN registry (number: ISRCTN12243194; https://www.isrctn.com/).

**Figure 2:**
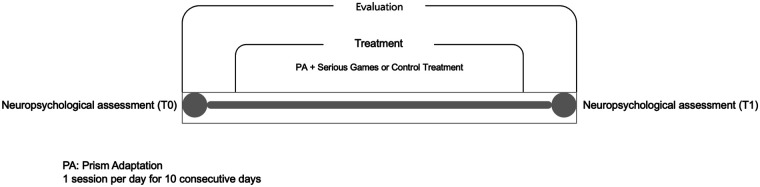
Experimental set up.

Figure 2 summarizes the experimental set up.

### Analyses

Statistical analyses were conducted using the Statistical Package for the Jamovi.

In each experimental group, paired *t*-tests were used to test for significant differences of T1 vs. T0 scores of the cognitive tests. The effect sizes for pre-test –post-test control group designs were estimated, based on the mean pre–post change in the treatment group minus the mean pre–post change in the control group, divided by the pooled pretest standard deviation (29). Unpaired *t*-tests were used to assess the difference in the improvement between groups.

Correlation analyses were applied between cognitive scores at T1 and scores of the Barthel Index at T1 and the corresponding correlations coefficients were compared between experimental and control group.

Regression analyses were applied on the average scores of the serious games across the ten sessions and of the neuropsychological tests at T1, in order to identify which serious games predicted the scores of the drawing tests and of spatial attentional tests (Albert's line cancellation and lime bisection) at T1.

To further clarify the impact of neglect (i.e., deficit of attention and representation of contralesional space) on visual-construction, additional regression analyses were made considering the scores at T1 of Freehand copy of drawings and Copy of drawings with landmarks tests as the dependent variable and the presence/absence of neglect as evaluated with Albert's cancellation and line bisection tasks as covariate.

All analyses were carried on test scores corrected for age and education according to parameters set in the published tests standardization.

*p*-values < 0.05 were considered statistically significant.

The experimenter who conducted the analyses on T1 vs. T0 effects was blinded to groups. The experimenter who conducted the regression analyses to identify which serious games predicted the scores of the drawing tests and of neglect tests at T1 could not blinded since these analyses could only be made on variables pertaining to the experimental group.

## Results

### Test scores at baseline in experimental and control group

Baseline (T0) performances in the neuropsychological tests and at the Barthel Index were compared to ensure that there was no statistical difference in the starting clinical conditions between the two groups of patients. Indeed, all scores at T0 were similar in the experimental vs. control group (unpaired *t*-tests: *p* > 0.05).

Ten control (67%) and 6 experimental (40%) patients had pathological scores at the Freehand copy of drawings (chi^2^ = 2.1, *p* > 0.05), 12 control (80%) and 13 experimental (87%) patients had pathological scores at the Copy of drawings with landmarks task (chi^2^ = 0.2, *p* > 0.05).

Nine control (60%) and 5 experimental (83.3%) patients had pathological scores at the Albert's line cancellation test (chi^2^ = 1.2, *p* > 0.05), 7 control (47%) and 2 experimental (13.3%) patients had pathological scores at Line bisection test (chi^2^ = 2.5, *p* > 0.05).

### Differences from T0 and T1 of the visual-constructive test scores

**Figure 3:**
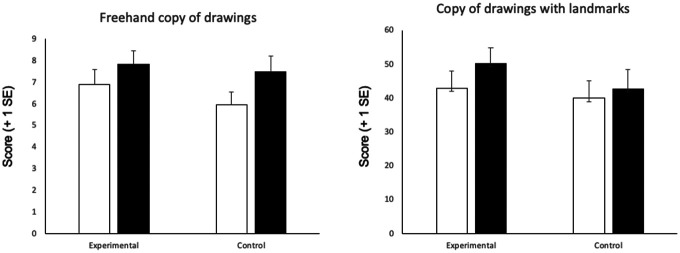
Average scores in the freehand copy of drawings and copy of drawings with landmarks tests at T0 (white columns) and T1 (black columns) in the experimental vs. control group. Error bars indicate standard error of mean.

Figure 3 shows the average corrected scores of the two drawing tests at T0 and T1 times in patients of the experimental and of the control group.

When analyzing the Freehand copy of drawings test, there was an improvement of the scores at T1 vs. T0 in patients of both the experimental (*t* = −2.7; *p* = 0.01; *d* = −0.7) and the control group (*t* = −2.7; *p* = 0.01; *d* = −0.7). Eight patients of the experimental and 10 patients of the control group improved from T0 to T1 assessments. The difference in the improvement between groups was not significant (*t* = 1.05; *p* = 0.3; *d* = 0.4).

When analyzing the Copy of drawings with landmarks test, there was a statistically significant improvement of the scores at T1 vs. T0 in patients of the experimental (*t* = −3.1; *p* = 0.007; *d* = −0.8) but not in those of the control group (*t* = −0.8; *p* = 0.4; *d* = −0.2). Twelve patients of the experimental and 9 patients of the control group improved from T0 to T1 assessments. The difference in the improvement between groups was not significant (*t* = −1.1; *p* = 0.2; *d* = −0.4).

In the experimental group, the group effect size on the post–pre comparison was 0.36 in the Copy of drawings with landmark test.

There was not any gender specificity of the observed effects in the two tests.

### Differences from T0 and T1 of functional scale and of spatial attentional test scores

Table 2 reports average scores of Barthel Index and of the two spatial attentional tests (Albert's line cancellation and line bisection) at T0 and T1 times in patients of the two groups.

**Table 2:** Mean and standard deviations of spatial attention test scores and Barthel Index scores at T0 and T1 in patients of the experimental and control group.

Cognitive test scores	Prisms ± Serious Games Group		Control Group	
	T0	T1	T0	T1
Line Bisection	5.9 ± 3.4	6.4 ± 3.2	3 ± 3.4	5.3 ± 3
Albert's line cancellation (left space)	12.4 ± 8.2	15.4 ± 5.6	11.8 ± 6.9	12.7 ± 7.3
Barthel Index	29.3 ± 25.6	39 ± 30.1	34 ± 26.6	50.6 ± 29.2

Barthel Index scores at T0 improved at T1 evaluation in the control group (*p* < 0.01; *d* = −0.9) and showed a trend towards improvement in the experimental group (*p* = 0.07; *d* = −0.5). The difference in the improvement between groups was not significant (unpaired *t*-tests: *t* = −1.02; *p* = 0.3, *d* = −0.4).

When looking at spatial attentional tests, Albert's line cancellation scores for the left space at T0 showed a trend towards improvement from T0 to T1 in the experimental (*t* = −2.08; *p* = 0.05; *d* = −0.5) but not in the control group (*t* = −0.6; *p* = 0.5; *d* = −0.1). The difference in the improvement between groups was not significant (unpaired *t*-tests: *t* = 1.05; *p* = 0.3, *d* = 0.4).

Line bisection test scores at T0 did not change at T1 in the experimental (*t* = −0.8; *p* = 0.4; *d* = −0.2), while they showed a significant improvement in the control group (*t* = −2.4; *p* < 0.05; *d* = −0.6). The difference in the improvement between groups was not significant (unpaired *t*-tests: *t* = −1.6; *p* = 0.1, *d* = −0.6).

### Correlation between improvement in spatial attention and improvement in visual-constructive abilities

In the experimental group, T1-T0 difference scores of both copy of drawings tests did not correlate with T1-T0 difference scores of the two spatial attentional tests (Line bisection and Albert's line cancellation) for assessing neglect.

**Figure 4:**
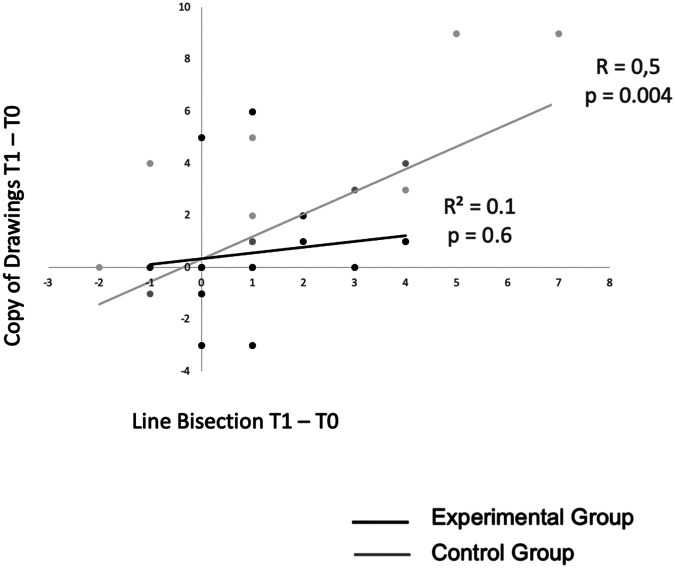
Correlation between T1-T0 difference scores of the copy of drawing and line bisection tests in the experimental vs. control group.

In the control group, T1-T0 difference scores of the freehand copy of drawing test significantly correlated with T1-T0 difference scores of the line bisection test (*R* = 0.6; *p* = 0.01) (Figure 4).

This result suggests that the magnitude of improvement in visual-constructive tests induced by prism adaptation in the experimental group is not correlated with the magnitude of improvement of contralesional spatial attention deficits (i.e., spatial neglect). Therefore, in the experimental group the mechanisms of improvement of neglect and of visual-constructive deficits probably follow different pathways.

### Correlation between cognitive test scores at T1 and Barthel Index scores at T1

Correlation analysis between Freehand copy of drawings and Barthel Index scores at T1 was significant in the experimental (*R* = 0.65; *p* = 0.009) but not in the control group (*R* = 0.29; *p* = 0.31). The slopes of the curves were not statistically different in the experimental vs. control group (*z* = 1.16; *p* = 0.1).

Correlation analysis between Copy of drawings with landmarks and Barthel Index scores at T1 was significant in the experimental (*R* = 0.58; *p* = 0.02) and tended towards significance in the control group (*R* = 0.48; *p* = 0.07). The slopes of the curves were not statistically different in the experimental vs. control group (*z* = 0.32; *p* = 0.3).

**Figure 5:**
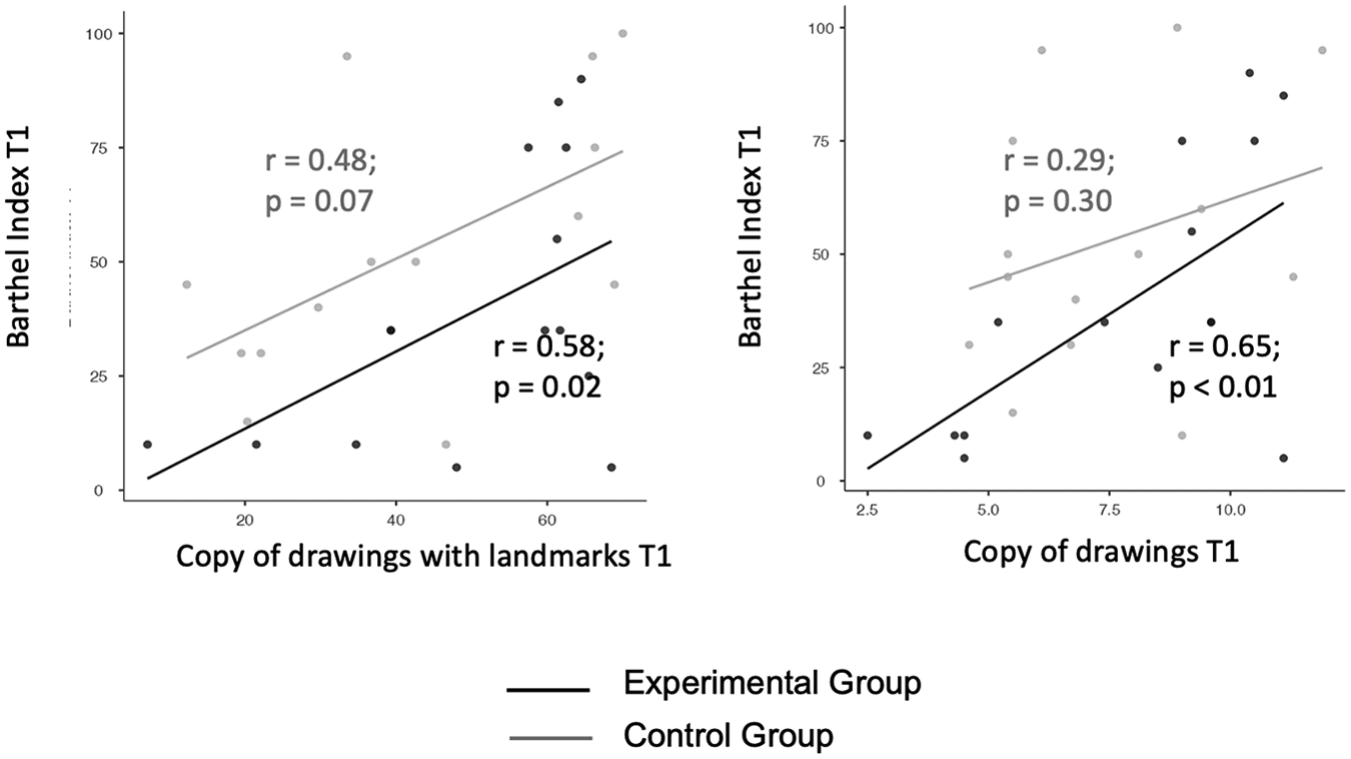
Correlation between T1 scores of the copy of drawings tests and T1 Barthel Index scores in the experimental vs. control group. Left panel: copy of drawings with landmarks. Right panel: freehand copy of drawings.

These results suggest that rehabilitation of visual constructive abilities generalizes to functional daily life activities, especially when the digital treatment is applied (Figure 5).

*Regression analyses to identify which serious games predicted the scores of the drawing and neglect tests at T1* .

Table 3 reports average scores of each serious game across the ten experimental sessions in patients of the experimental group.

**Table 3:** Average scores of the serious games across the ten sessions in patients of the experimental group.

Serious game	Sessions									
	1	2	3	4	5	6	7	8	9	10
Go no-Go	36	33.2	35.6	39	39.8	41.8	39.9	43.2	45.4	41.2
Beware of the Bomb	14.1	15.6	18	20	17.5	20.9	17.7	21.9	21.2	21.1
The Cafè	8.8	13.7	16.8	19	20.1	16.6	21.6	20.2	19.6	22.9
Visual Search	10.9	10.8	13.4	15	15.3	13.3	13.9	15.2	15	14.9
Reverse Order	4.6	6.8	6.1	6.7	7	7.9	6.6	8	9.9	8.9
Calculation	6.9	8.4	10.6	9.6	10.1	11	10.1	9.9	10.6	10.6
Semantic Associations	5.3	4.8	5.1	5.4	5.9	6.4	6.4	6.4	5.9	6.8

T1 performance at the Freehand copy of drawings test was predicted by the average scores of serious games “Beware of the bomb” (*R*^2^ = 0.8; beta = 0.2; SE = 0.02; *t* = 6.1; *p* < 0.001), “The cafè” (*R*^2^ = 0.6; beta = 0.2; SE = 0.04; *t* = 4; *p* = 0.002), “Reverse Order” (*R*^2^ = 0.4; beta = 0.4; SE = 0.1; *t* = 3.1; *p* = 0.009), “Visual Search” (*R*^2^ = 0.6; beta = 0.3; SE = 0.06; *t* = 4.2; *p* = 0.001).

T1 performance at the Copy of drawings with landmarks test was also predicted by the average scores of the serious games “Beware of the bomb” (*R*^2^ = 0.6; beta = 0.9; SE = 0.2; *t* = 3.9; *p* = 0.002), “The cafè” (*R*^2^ = 0.6; beta = 1.2; SE = 0.3; *t* = 3.9; *p* = 0.002), “Reverse Order” (*R*^2^ = 0.5; beta = 2.6; SE = 0.8; *t* = 3.3; *p* = 0.006), “Visual Search” (*R*^2^ = 0.6; beta = 1.7; SE = 0.4; *t* = 4.1; *p* = 0.001).

In sum, the serious games targeting visuo-spatial attention, visual search and planning do predict the scores of both visual-constructive tests. On the other hand, the serious games targeting inhibition (go/no go), semantic associations and verbal working memory/calculation do not predict the scores of any of the visual-constructive tests.

T1 performance at the Albert's line cancellation test (number of cancellations in the left space) was predicted by the average scores of three serious games: “beware of the bomb” (*R*^2^ = 0.31; beta = 0.23; SE = 0.10; *t* = 2.3; *p* = 0.03); “the cafè” (*R*^2^ = 0.48; beta = 0.37; SE = 0.11; *t* = 3.34; *p* = 0.006); “visual search” (*R*^2^ = 0.35; beta = 0.45; SE = 0.17; *t* = 2.55; *p* = 0.02).

T1 performance at the line bisection test was predicted by the average scores of two serious games: “beware of the bomb” (*R*^2^ = 0.33; beta = 0.12; SE = 0.05; *t* = 2.43; *p* = 0.03); “reverse order” (*R*^2^ = 0.29; beta = 0.34; SE = 0.15; *t* = 2.2; *p* = 0.04).

### Regression analyses to measure the impact of spatial neglect on visual-constructive tests

T1 scores of the Freehand copy of drawings test were not predicted by the presence of spatial neglect (*R*^2^ = 0.3; beta = −1.7; SE = 1.1; *t* = −1.5; *p* = 0.1), without difference between experimental and control group (*t* = −1.5; *p* = 0.1).

T1 scores of the Copy of drawings with landmarks test were instead strongly predicted by the presence of spatial neglect (*R*^2^ = 0.5; beta = −30.3; SE = 7.5; *t* = −4; *p* < 0.001), without difference between the experimental and control group (*t* = 0.5; *p* = 0.6).

These findings show how the presence of spatial neglect negatively interferes with rehabilitation of the copy of drawings with landmarks test, without difference between experimental and control groups.

**Figure 6:**
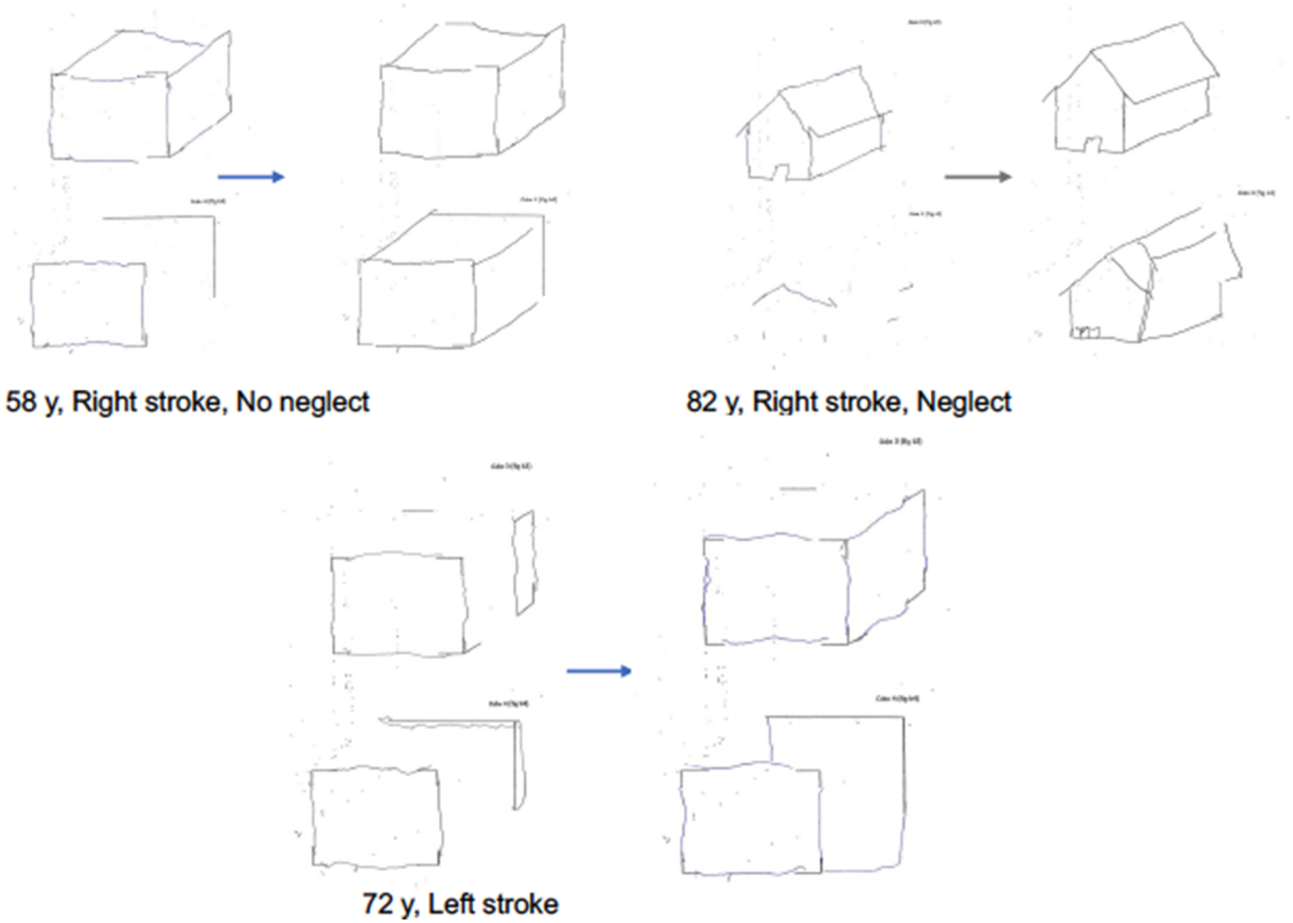
Examples of patients' drawings in the copy of drawings with landmarks test at T0 and T1 in the experimental group. Patient with right stroke, without neglect. Patient with right stroke, with visuospatial neglect. Patient with left stroke.

Figure 6 shows an example of copy of drawing with landmarks in three representative patients of the experimental group: a right brain damaged patient with left neglect (a), a right brain damaged patient without neglect (b), a left-brain damaged patient (c).

## Discussion

### Main findings

The main results of this study show that a therapeutic approach combining digitized visuomotor adaptation to optical prisms with cognitive training through serious games, is superior to standard rehabilitation therapy for improving some visual-constructive deficits following stroke. The most significant advantage of this approach is shown for deficits assessed with the copy of drawings with landmarks test.

Improvements in both the freehand copy of drawings and the copy of drawings with landmarks tasks following a 10-day rehabilitation protocol were found to predict better performance in activities of daily living (ADLs) for patients in the experimental group, while this correlation was less strong in the control group.

Furthermore, the improvement in drawing tasks in the experimental group was predicted by the performance of stroke patients in specific serious games that are part of the therapeutic approach. These games target cognitive skills such as sustained and divided visual attention, planning, and visual search.

The serious games also showed specificity in relation to two tests used to measure contralesional spatial attentional deficits (i.e., neglect). Specifically, scores on the Albert's cancellation test were predicted by performance in serious games targeting sustained and divided attention, visual search, and motor planning, while scores on the line bisection test were predicted by performance in games that focus on sustained attention and spatial working memory along the horizontal plane.

These results complement those reported in a previous study, showing how a treatment using the same combination of prism adaptation and serious games improves measures of attention, verbal and spatial working memory and inhibition abilities in stroke patients (16).

### Interpretation of results as related to improvement of contralesional spatial attentional deficits

At first glance, one might attribute the improvements in visual-constructive deficits observed in this study to a reduction in visuospatial left attentional deficits (i.e., neglect) following prism adaptation.

Indeed, prism adaptation is a method that has been shown to facilitate the rehabilitation of neglect (although its validity in this area has recently been questioned; see (30, 31), and spatial neglect can impair visual-constructive tasks since difficulties with visual scanning and spatial representation are common to both spatial neglect and constructional apraxia (1, 6, 32).

Consistent with this, neglect was a predictor of performance on at least one of the visual-constructive tasks (copy of figures with landmarks). However, neglect had a similar impact on visual-constructive abilities in both the experimental and control groups, and most patients with neglect were in the control group. Moreover, the lack of correlation between the magnitude of improvement in neglect and that in visual-constructive tasks in the experimental group suggests that the observed improvements of visual-constructive tasks in the experimental group are not solely due to the impact of prism adaptation on neglect in patients with right brain damage.

Therefore, beyond the rehabilitation of neglect *per se*, our findings suggest that the therapeutic protocol applied in the experimental group likely operates through mechanisms that go beyond the reshaping of visuospatial attention alone.

### Interpretation of results in terms of reshaping of cortical circuits

Recent findings showed that prism adaptation can modulate cortical plasticity, with hemispheric specificity linked to the direction of the visuomotor deviation.

Increased cortical excitability has been documented in the hemisphere ipsilateral to the visual field deviation induced by prisms (13, 17, 33). Previous research has also shown that these neurophysiological effects translated into improvements in specific cognitive functions, including language following left hemispheric modulation through leftward prisms (34) and memory following either right or left hemispheric modulation through rightward or leftward prisms (35). In line with this mechanism of action, the present study applied prism adaptation to stroke patients with deviations ipsilateral to the affected hemisphere (i.e., right deviation in RBD and left deviation in LBD patients). The underlying idea is that prism adaptation enhances cortical excitability in the residual neurons of the affected hemisphere, an effect shown to be comparable to that induced by brain stimulation techniques (13). This increased excitability likely makes neural circuits more receptive to cognitive training through serious games that target cognitive processes involved in visual-constructive abilities.

An interpretation of the improvement in drawings tasks based on hemispheric modulation of excitability of parieto-frontal-temporal networks (2, 36) is consistent with the heterogeneous nature of visual-constructive disorders, which involve a wide network of brain regions subserving cognitive, perceptual and motor processes essential for accurate copying and drawing (6).

### The role of digital cognitive training in rehabilitation of visual-constructive abilities

The logic of serious games is to train specific cognitive functions following prism-induced neuromodulation. This is in line with TMS and tDCS studies combining brain-stimulation induced neuromodulation with digital cognitive training [e.g., (37)].

Since the prism adaptation training procedure prevents concurrent cognitive training through serious games and since cognitive training prior to prism adaptation could induce metaplastic effects leading to paradoxical modulations of brain excitability (13), the device Mindlenses Professional used in the present study applies cognitive training immediately following prism adaptation.

In relation to the cognitive processes involved, it is noteworthy that the serious games whose scores most strongly correlate with improvements in visual-constructive tasks are those that target visual search, visuospatial attention and planning—cognitive functions most associated with right hemispheric activation. Indeed, the majority of patients recruited for the present trial had right hemispheric lesions and the drawing tasks investigated here are closely linked to right hemispheric functional components.

Sustained attention is a key resource function for maintaining a cognitive set and it plays a critical role in performance on figure copying tasks (38, 39). Additionally, the ability to copy a complex figure involves visuomotor transformation, which requires both visual perception and eye-hand coordination. This may explain why visuomotor adaptation via prisms likely impacts visual-constructive abilities, particularly when paired with cognitive training focused on visual search and attention.

Consistently with this interpretation, recent findings showed that prism adaptation can indeed improve constructional deficits (40), left hyperschematia (41) and copying of figures, along with measures of functional outcome, when combined with specific cognitive trainings (42). The relationship between visuomotor adaptation and constructional apraxia is also supported by evidence linking the condition to deficits in remapping visual information across saccades (43).

The results of digital training on spatial attention also suggest that different cognitive components are involved in each test and that specific digital training can be used to target these components. The Albert's cancellation task, which requires exploration of targets (lines) in space and planning movements to bisect the lines, emphasizes exploratory, planning, and visual search abilities. Consistent with this, the task was specifically modulated by games targeting sustained and divided attention, visual search, and motor planning. On the other hand, the line bisection task is more related to the movement of spatial attention along the horizontal plane, requiring the subject to focus on a centrally located line to identify its bisection point. Consistent with this, the task was more modulated by games targeting sustained attention and spatial working memory along the horizontal plane.

These specific effects of cognitive training following neuromodulation-induced by PA argue against unspecific arousal effects of serious games, and they rather suggest the importance of using serious games targeting specific components related to the functions to be rehabilitated.

### The role of right vs. left hemispheric components in visual-constructive abilities

The most significant difference between the experimental and control treatment concerned the task of copying of figures with landmarks. Differently from the freehand figure copy task, this test provides the patient with landmarks on which to base the planning of the movements necessary for copying the figure. Thus, the patients may use the provided points as a foundation for planning and executing their copying strategy. It has been argued that the presence of landmarks could offer a structured and organized approach to figure copying, that could facilitate patients with left brain damage due to their deficits in organizing and planning tasks (44). This hypothesis was not confirmed by larger group studies (4) and the limited number of patients with left brain damage recruited in the present trial does not allow to address this issue. However, it is worth noting that 5 out of the 7 patients with left brain damage in the present trial were in the experimental group and all of them improved in the figure of copying task with landmarks from T0 to T1.

Regardless of the contribution of left hemispheric components in the rehabilitation of this specific test, the advantage of the approach of combining prisms with serious games suggests that specific rehabilitation of visuomotor and planning components gives a greater advantage when the task requires to rely on programming elements in figure copying.

In addition to these considerations, it is also worth noting that the copy of drawings with landmarks was also the test with the higher number of pathological scores in the present series, a factor that could facilitate the emergence of differences between the experimental and control treatment.

Qualitative analysis of the characteristic of the drawings of patients is consistent with previous literature in showing how the drawings of right-brain damaged patients with constructional apraxia are characterized by lack of accurate spatial relations between objects items, while drawings of the left-brain damaged patients are more characterized by oversimplification of figures and by perseveration on items suggestive of planning deficits (1). At a qualitative analysis, the experimental treatment seems to modulate both deficits.

### Generalization of the improvements to activities of daily living

An objective of the present study was to test whether cognitive effects in copy of drawings tests transfer to functional activities. In fact, results suggest that it is mainly the experimental treatment with prism adaptation and serious games that shows an effect of generalization, transferring its cognitive effects in daily life activities of stroke patients.

This result confirms previous observations that showed a generalization of cognitive effects related to working memory following this kind of treatment approach (16).

We suggest that the generalizability of rehabilitation of one task to global impact on functional independence using prisms and serious games is related to their mechanism of action. In fact, a mechanism of action related to modulation of the plasticity of large brain networks can favor modulation of functional components that are transmodal rather than task-specific, which could then improve a wide range of cognitive and motor functions.

### Limitations

The present study had some limits. The balance of patients in the two randomized groups could have been better controlled for some variables, such as education levels, that in this case clearly advantaged the control group (i.e., high education is a factor that facilitates cognitive recovery). To address this bias, we analysed test scores corrected for age and education levels according to correction tables available for each of the tests employed.

The sample size was determined considering the magnitude of effects of prism adaptation on cognitive functions in previous studies. This could have led to underestimation of the number of patients to be recruited, increasing the risk of type II error.

Although randomization was done to include patients in experimental and control groups, patients could not be blinded to the intervention received, due to the nature of the experimental treatment that makes use of a recognizable wearable device and a software.

The results presented in this paper refer to tests measuring visual-constructive, spatial attentional and functional abilities. These were specifically investigated because of the relationship linking visual-constructive with spatial attentional abilities, that are modulated by both prisms and the serious games that are part of the device. Results of all other tests have been preliminarily published in a public registry.

The number of left-brain damaged patients was too low to allow for separate analyses related to hemispheric differences in visuo-constructional deficits.

Rehabilitation of the control group was the standard one. In other clinical trials, currently ongoing, the control group is also treated with serious games alone, which assures better balancing between groups.

## Conclusions

The results of the present study could have practical and research implications.

From a research perspective, the results extend current knowledge on the cognitive mechanisms subserving impairment of visual-constructive abilities following stroke.

From a clinical point of view, they suggest that digital prism adaptation and digital cognitive training through specific serious games could be a new tool for cognitive neurorehabilitation in stroke patients, producing its effects in a ten-session short term time window.

## Data Availability

The data will be made available upon request to the corresponding author.
